# Race, Healthcare, and Health Disparities: A Critical Review and Recommendations for Advancing Health Equity

**DOI:** 10.5811/westjem.58408

**Published:** 2023-08-08

**Authors:** Wendy L. Macias-Konstantopoulos, Kimberly A. Collins, Rosemarie Diaz, Herbert C. Duber, Courtney D. Edwards, Antony P. Hsu, Megan L. Ranney, Ralph J. Riviello, Zachary S. Wettstein, Carolyn J. Sachs

**Affiliations:** * Center for Social Justice and Health Equity, Department of Emergency Medicine, Boston, Massachusetts; † Harvard Medical School, Department of Emergency Medicine, Boston, Massachusetts; ‡ Tampa General Hospital, Tampa, Florida; § University of California-Los Angeles, Department of Emergency Medicine, Los Angeles, California; ∥ University of Washington School of Medicine, Department of Emergency Medicine, Seattle, Washington; ¶ Washington State Department of Health, Tumwater, Washington; # Samford University, Moffett & Sanders School of Nursing, Birmingham, Alabama; ** Trinity Health Ann Arbor Hospital, Department of Emergency Medicine, Ypsilanti, Michigan; †† Yale University, Yale School of Public Health, New Haven, Connecticut; ‡‡ University of Texas Health San Antonio, Department of Emergency Medicine, San Antonio, Texas; §§ University of Washington School of Medicine, Department of Emergency Medicine, Seattle, Washington; ∥∥ Ronald Reagan-UCLA Medical Center and David Geffen School of Medicine at University of California-Los Angeles, Department of Emergency Medicine, Los Angeles, California

**Keywords:** health disparities, social determinants of health, structural racism, implicit bias

## Abstract

An overwhelming body of evidence points to an inextricable link between race and health disparities in the United States. Although race is best understood as a social construct, its role in health outcomes has historically been attributed to increasingly debunked theories of underlying biological and genetic differences across races. Recently, growing calls for health equity and social justice have raised awareness of the impact of implicit bias and structural racism on social determinants of health, healthcare quality, and ultimately, health outcomes. This more nuanced recognition of the role of race in health disparities has, in turn, facilitated introspective racial disparities research, root cause analyses, and changes in practice within the medical community. Examining the complex interplay between race, social determinants of health, and health outcomes allows systems of health to create mechanisms for checks and balances that mitigate unfair and avoidable health inequalities.

As one of the specialties most intertwined with social medicine, emergency medicine (EM) is ideally positioned to address racism in medicine, develop health equity metrics, monitor disparities in clinical performance data, identify research gaps, implement processes and policies to eliminate racial health inequities, and promote anti-racist ideals as advocates for structural change. In this critical review our aim was to (a) provide a synopsis of racial disparities across a broad scope of clinical pathology interests addressed in emergency departments—communicable diseases, non-communicable conditions, and injuries—and (b) through a race-conscious analysis, develop EM practice recommendations for advancing a culture of equity with the potential for measurable impact on healthcare quality and health outcomes.

## INTRODUCTION

Social determinants of health (SDoH) as defined by the US Centers for Disease Control and Prevention (CDC) are the conditions in which people live, learn, work, and play that are determined by the distribution of money, power, and resources and that affect a wide range of health and quality-of-life risks and outcomes.^1^ Influenced by the social construct of race, SDoH exert disparate impacts on the health of subpopulations. Economic disparities disproportionately place Black, indigenous, and people of color (BIPOC) within zones marked by substandard health promotion and excessive health risks. The compounding nature of adverse SDoH, such as housing instability, food insecurity, poor healthcare access, and hazardous exposures, has serious health implications. Health disparities are the profound downstream effect of the socioeconomic disadvantages that BIPOC endure under the moniker *structural racism*.

In addition to structural racism, *implicit bias*—defined as unconscious attitudes, positive or negative, toward a person, group, or idea—often leads to differential treatment based on perceived race.^2^^,^^3^ Implicit bias further restricts quality healthcare as a separate factor above and beyond inequities of structural racism. Emergency department (ED) data indicates that Black (vs White) patients have longer treatment wait times,^4^ longer lengths of stay,^5^ and lower triage acuity levels.^6^ Additionally, Black ED patients have a 10% lower likelihood of admission and 1.26 times higher odds of ED or hospital death than White patients.^7^ Research also suggests that physicians’ own implicit racial biases may contribute to disparities in healthcare quality and delivery.^8^^–^^10^

In this critical review we explore the complex effects of race, implicit bias, and structural racism on SDoH, healthcare quality and, ultimately, health outcomes. Although not intended as a comprehensive literature review on health disparities, this exercise informs a conceptual framework through which actionable steps and practice recommendations for emergency medicine (EM) are proposed as one part of a larger systemwide effort that requires thoughtful action and transformative policy to dismantle the hard-wired inequities of structural racism and advance health equity.

## METHODS

### Critical Review Methodology

We conducted a broad-scope critical review of the extant health disparities literature across three areas of clinical pathology interest: communicable diseases; non-communicable conditions; and injuries. The review was conducted through a race-conscious lens to examine the impact of race on health outcomes and inform a conceptual framework for the development of actionable steps and practice recommendations.

Critical reviews include “a degree of analysis and conceptual innovation” resulting in a product capable of launching a new phase of evaluation.^11^ According to Grant and Booth, the critical review does not call for a systematic evaluation of all the literature related to a topic, but rather the emphasis is on the contribution of each piece of evidence included to the review’s conceptual product.^11^ As described by the Search, Appraisal, Synthesis, and Analysis framework, critical reviews are designed to identify key findings in the field of interest (health disparities literature), evaluate the evidence in accordance with its contribution (racial health disparities attributable to SDoH), synthesize the evidence in organized fashion (clinical pathology interests relevant to EM), and provide a conceptual output of analysis that contributes to the literature (actionable steps and practice recommendations).^11^

In this review we aimed to examine racial health disparities through the SDoH model and apply socioenvironmental theory^12^ and resource deprivation theory^13^ as race-conscious filters through which racial disparities data is analyzed and synthesized (Table 1). The analysis informed the conceptual framework through which we developed and propose actionable steps and practice recommendations.

**Table 1.** Race-conscious analysis tools employed in critical review.

Socioenvironmental theory^12^	Resource deprivation theory^13^
*Socioenvironmental theory* holds that racial residential segregation is central to racial and ethnic health disparities. According to this theory, racial/ethnic minority groups have considerably different levels of health risk due to the multiple social and environmental factors that detrimentally impact their health within the context of longstanding residential segregation and its deeply rooted socioeconomic disadvantages.	*Resource deprivation theory* holds that the longstanding deprivation of resources experienced by racial/ethnic minority groups is central to racial and ethnic disparities. Due to chronic deprivation, racial/ethnic minority groups lack the necessary infrastructure to support health. Resources are not restricted to material possessions; they include education, employment, housing, neighborhood safety, and psychological wellbeing. According to evidence-based interpretations of this theory, gap closure cannot be achieved through equal distribution of resources, but rather targeted differential distribution of resources that levels the playing field for racial/ethnic minority groups.

## RESULTS

### Communicable Diseases

#### HIV/AIDS

Racial and ethnic disparities in the incidence and prevalence of HIV infection and AIDS have been documented in the US since the 1980s.^14^ Despite prevention, identification, and treatment advances, Black-White and Hispanic-White disease incidence disparities have increased since 1984. In 2013, Blacks and Hispanics accounted for 46% and 21% of new HIV infections and 49% and 20% of new AIDS diagnoses despite representing 12% and 16% of the total US population, respectively.^14^ Although HIV incidence rates have improved in recent decades, Blacks and Hispanics have benefitted less from antiretroviral therapy advancements.^15^ Incidence rates (IR) have declined with the advent of pre-exposure prophylaxis (PrEP); however, PrEP usage remains disparately low among Black (5.9%) and Hispanic (10.9%) adults with an indication as compared to Whites (42.1%).^16^^,^^17^

*ED Actionable Steps:* Increase access to HIV testing and referrals to PrEP and post-exposure prophylaxis.

#### Viral Hepatitis

Hepatitis C virus (HCV) is the leading cause of liver disease-related death in the US.^18^ Racial disparities in disease prevalence exist at a rate greater than twice that of Whites; Blacks in the US have the highest prevalence ratio (PR) of disease (PR 2.29, 95% confidence interval [CI] 1.94–2.70).^18^ Rates of treatment for chronic hepatitis C are also higher among Whites as compared to Black, Hispanic, and Asian individuals.^19^ Direct-acting antivirals (DAA) became available in 2014 and are achieving greater than 90% cure rates.^20^ Early research found that Black and Hispanic patients were less likely than Whites to benefit from DAA initiation (adjusted rate ratio [aRR] 0.7, 95% CI 0.7–0.8 and 0.8, 95% CI 0.7–0.9, respectively).^21^ Follow-up data from a national cohort found that these racial-ethnic gaps had closed by 2016; however, more recent data is needed to determine whether equitable access has persisted beyond initial evidence-driven efforts.^20^

*ED Actionable Steps:* Increase access to HCV testing and referrals to DAA treatment.

#### Sexually Transmitted Infections

Disparities in sexually transmitted infections (STI) have been described extensively in the literature. Rates of primary and secondary syphilis, HIV/AIDS, chlamydia, and gonorrhea among Blacks range from 5.4 to 17.8 times the rates among Whites in the US.^22^ The SDoH associated with increased STI prevalence have been discussed extensively, ranging from inequities in healthcare, income, incarceration, residential segregation, and substance use, among others.^23^^,^^24^ Importantly, prevalence must be interpreted within the context of STI screening, the odds of which are higher among Black and Hispanic women than their White counterparts (adjusted odds ratio [aOR] 2.56, 95% CI 2.60–3.10 and 1.42, 95% CI 1.39–1.46, respectively).^25^

*ED Actionable Steps:* Increase access to STI testing and ED-based treatment.

#### Diarrheal Disease

An estimated 500,000 cases of shigellosis occur annually in the US.^26^ Incidence rates of infection per 100,000 are greatest among Black (7.2) and Hispanic (5.6) individuals as compared to Whites (2.6).^26^ Despite the preventable nature of shigellosis, an analysis of over 25,000 laboratory-confirmed cases reported to the CDC found a strong association between its incidence and residence in areas marked by US Census Tract-level poverty and household crowding. Racial and ethnic IR disparities, however, persisted even after controlling for these socioeconomic indicators,^26^ and the rates of severe infection among adults are highest among Black persons.^27^ Similarly, Black (vs non-Black) infants <6 months in age had higher rates of diarrhea-associated hospitalizations that persisted even after the introduction of the rotavirus vaccines in 2006.^28^

*ED Actionable Steps:* Educate patients and parents about transmission mechanisms and mitigation strategies (eg, hand hygiene, low-cost water treatment options, vaccination), and consider offering vaccination in the ED when necessary and reasonable.

#### Pandemic Respiratory Viral Infection

Disparities exist among pandemic respiratory viral infections, including influenza H1N1 and severe acute respiratory syndrome coronavirus 2 (SARS-CoV-2), resulting in higher disease incidence and mortality among minority groups.^29^^–^^31^ Coronavirus disease 2019 (COVID-19) cases and hospitalization rates were 2.5-4.5 times higher among Black, Hispanic, and Native American populations than Whites. Through May 2021, COVID-19 deaths among Hispanic and Black populations were 17% and 10% greater, respectively, than expected by US population representation after controlling for age.^32^ Elevated COVID-19 infection and death rates have also been observed in socially disadvantaged counties with larger proportions of BIPOC.^32^^,^^33^ Among residents of a predominantly Black and Hispanic COVID-19 hotspot, very high and disparate positivity rates were observed among Black (68.5%) and Hispanic (65.3%) patients as compared to Whites (53%).^34^ Higher hospitalization rates for Blacks (60.2%) and Hispanics (62.3%) as compared to Whites (47.7%) were also observed, although there were no differences in admission rates to the intensive care unit.^34^

Mortality rates among COVID-19 inpatients also show BIPOC disparities.^35^^,^^36^ Recent CDC data shows higher mortality risk ratios for Native Americans (2.4), Hispanics (2.3), and Blacks (1.9) compared to Whites.^37^ There are several reasons cited to explain the higher out-of-hospital mortality rates, disease burden, and severity of illness among BIPOC.^36^^,^^38^^–^^40^ Several authors have concluded that population-based disparities in COVID-19 hospital mortality are best explained by differential disease incidence, prevalence of comorbid conditions, and socioeconomic marginalization among Black and Hispanic individuals.^34^^,^^39^^,^^40^

Overall racial and ethnic disparities in COVID-19 risk, severity, morbidity, and mortality arise from a combination of social, economic, and health determinants.^36^^,^^38^ Due to economic strain, BIPOC are more likely to live in crowded housing (multigenerational or communal households) and densely populated neighborhoods. They are also more likely to work in consumer-facing public service industries and rely on public transportation, increasing their exposure risk. Additionally, higher rates of comorbidities (eg, heart disease, diabetes, hypertension, and obesity) increase BIPOC’s risk for severe COVID-19 disease. Barriers to health insurance and health services limit access to treatments and to accurate knowledge regarding SARS-CoV-2 transmission, prevention strategies, disease symptoms, and reasons for seeking care.^41^^–^^43^ Interestingly, despite the positive impact of Medicaid expansion on healthcare access, mortality, and disparities, one study failed to find an association between COVID-19 mortality and expansion vs non-expansion,^44^^,^^45^ likely reflecting a benefit negated by the heightened social risk of structural racism.

Disparities in vaccination coverage were evident by the end of April 2021. When all adult age groups were eligible, vaccination rates among Black (46.3%) and Hispanic (47.3%) adults were lower than among Whites (59%) and Asians (69.6%).^46^ Despite policies to ensure equitable COVID-19 vaccine access, vaccination hesitancy—originating from distrust in the medical establishment and resulting from longstanding systemic racism in healthcare and research—required community partnerships and concerted efforts by trusted sources of information to overcome the slower rates of vaccination among BIPOC.^46^

*ED Actionable Steps:* Increase access to viral testing, educate patients and parents about transmission mechanisms and mitigation strategies (eg, masks, isolation, vaccination), and consider offering vaccination in the ED when necessary and reasonable.

### Non-Communicable Conditions

#### Acute Coronary Syndrome and Acute Myocardial Infarction

Disparities in acute coronary syndrome (ACS) care have been well-documented. Compared to White patients with door-to-balloon (DTB) times of 103.4 minutes, Black and Hispanic patients experience significantly longer DTB times (122.3 and 114.8 minutes, respectively).^47^ Over the last decade, DTB times have improved significantly across all groups; however, Black Americans have a lower likelihood of experiencing DTB times <90 minutes^48^ and have experienced only a modest decline in recurrent hospitalization for acute myocardial infarction (AMI) compared to Whites.^49^ Black patients experience worse AMI outcomes with a five-year mortality rate of 29% compared to 18% among Whites.^50^

*ED Actionable Steps:* Consider protocolized ED triage and early management of potential ACS/AMI-related complaints beyond chest pain.

#### Type 2 Diabetes Mellitus

Type 2 diabetes prevalence rates among Black (13.2%) and Hispanic (12.8%) Americans are similar and higher than rates among Whites (7.6%).^51^ Well-controlled glycemia and hospitalization rates, quality indicators, are both worse among Black patients (37.6% and 26.5%, respectively) compared to Whites (44% and 16.1%, respectively).^51^ The marker of glycemic control, hemoglobin A_1c_ (HgbA_1c_), is statistically worse among Black vs White patients (HgbA_1c_ 9.1 ± 2.9% vs. 8.5 ± 2.2%, *P* = 0.001).^52^ Black and Hispanic patients have higher odds of diabetes-related ED visits (odds ratio [OR] 1.84, 95% confidence interval [CI] 1.7–2.0 and 1.60, 95% CI 1.4–1.8, respectively) than Whites.^53^

*ED Actionable Steps:* Educate patients about the complications of poor glycemic control and consider navigation partnerships with primary care for expedited post-ED visit, outpatient follow-up of patients with diabetes-related chief complaints and complications.

#### Hypertension

Racial and ethnic disparities in hypertension are likely multifactorial related to upstream SDoH, including access to healthcare, affordable medications, low-sodium foods, and safe green spaces for physical activity.^54^ Unique to Black patients, race-consciousness significantly increases diastolic blood pressure (BP), and the self-perception of having a lower social standing as a function of race is associated with medication non-adherence and higher systolic BP.^54^ Research has also demonstrated that Black and Asian patients have higher odds of a high BP reading at their last clinic visit (OR 0.36, 95% CI 0.21–0.60 and 0.40, 95% CI 0.16–0.97, respectively) and Black and American Indian/Alaska Native patients have higher odds of an ED visit or hospitalization (OR 3.61, 95% CI 1.88–6.91 and 5.31, 95% CI 2.13–13.20, respectively).^55^

*ED Actionable Steps:* Educate patients about the complications of poor BP control and consider navigation partnerships with primary care for expedited post-ED visit, outpatient follow-up of patients with hypertension-related chief complaints and complications.

#### End-stage Renal Disease

Racial and ethnic disparities are profound in renal disease. Black patients experience higher IRs of end-stage renal disease (ESRD) in adolescence, greater probability of progression to advanced disease stages before initiation of dialysis, lower likelihood of peritoneal vs hemodialysis treatment, lower likelihood of transplant waitlist placement, and longer waiting times for transplantation.^56^ Pediatric nephrology registry data found that among children who progressed to ESRD, 41.8% of White children received transplants compared to 16.3% and 27% of Black and Hispanic children, respectively, and 70% of White children were transplanted within two years of waitlist placement compared to 44% of Black pediatric patients.^57^ Subsequent analyses confirm the persistence of these disparities with Black and Hispanic less likely than White children to receive preemptive transplants (8.7% and 14.2% vs 27.4%, respectively), and Black pediatric transplant recipients were less likely than White to experience allograft survival at five years (63% vs 80.8%, respectively).^58^

Similar disparities among non-White adult ESRD patients include lower rates of transplant referrals, delayed times to transplant waitlist placement, and longer wait times for transplant.^56^ National mortality statistics indicate Blacks experience significantly higher death rates from ESRD than Hispanic and White Americans (24.6 vs 11.1 and 12.1 age-adjusted death rate per 100,000, respectively).^59^

*ED Actionable Steps:* Advocate for increased access to dialysis, particularly for the uninsured, and consider navigation partnerships with nephrology and local dialysis centers for expedited post-ED visit, outpatient follow-up of patients with ESRD-related chief complaints and complications.

#### Obesity

As a risk factor for heart disease, type 2 diabetes, hypertension, and other chronic conditions, obesity poses a real challenge to population health management efforts. National data demonstrates that the highest prevalence of adult obesity occurs among Black Americans (38.4%) followed by Hispanics (32.6%) and Whites (28.6%).^60^ Much like hypertension, racial and ethnic disparities in obesity are multifactorial and require a multifaceted intervention to target social (food deserts), biological (hormone dysregulation secondary adverse childhood events), and behavioral (physical activity) determinants.^61^ Research has revealed a high burden of fast- food establishments within predominantly Black communities.^62^^–^^64^ Treatment disparities are also present with BIPOC demonstrating decreased responsiveness to weight-loss pharmacotherapy, decreased likelihood weight- loss center referral, and decreased likelihood of bariatric surgery.^65^

*ED Actionable Steps:* Consider partnerships with community programs focused on healthy lifestyle change and prescribe vouchers to patients whose health would benefit from weight loss.

#### Mental Health

Racial disparities in the management of psychiatric illness have also come to the forefront in recent years. Rates of depression treatment are lower among Black and Hispanic patients as compared to White patients, who are half as likely and a third as likely, respectively, to receive care than White patients.^66^ According to the CDC, Black adults had the highest rates of mental health-related ED visits in 2018-2020, had longer ED wait times, and were less likely to be admitted or transferred to another hospital.^67^ An analysis of national data found that Black patients presenting to the ED with a psychiatric emergency have a greater probability of chemical sedation than White patients.^68^^,^^69^ Additionally, single- and multisite studies have found that Black^69^^–^^71^ and Hispanic patients^71^ are more likely to be physically restrained in the ED than White patients.

*ED Actionable Steps:* Use an equity lens to conduct a thorough review of policies related to restraint use, consider protocolized screening and management of agitation inclusive of early oral medication and withdrawal treatment, and consider navigation partnerships with hospital-based and community-based counseling services.

### Injuries

#### Environmental Hazard-Related Injuries

Ambient fine-particulate matter exposure (PM 2.5) is a risk factor for a host of conditions including reactive airway disease, coronary artery disease, and cerebrovascular disease.^72^ The inequitable distribution of hazardous sites, namely industrial facilities, utilities, and landfills, is one of the greatest concerns in the field of environmental justice. Extensive literature has demonstrated that non-Whites are more likely to reside near stationary sources of PM, with Black Americans experiencing a higher burden of PM exposure than Whites and the general population.^73^

Racial disparities in hazardous exposure burden are not a recent phenomenon. The 1987 groundbreaking study that first exposed the disproportionate co-location of toxic waste sites and minority communities found that three of every five Black and Hispanic Americans lived in such conditions.^74^ The National Research Council conducted a study that observed greater prevalence of health problems—spontaneous abortions, birth defects, heart disease, gastric cancer, leukemia, and Hodgkin’s lymphoma—among those living in proximity to highly toxic chemicals and carcinogens (eg, benzene, polychlorinated biphenyls, mercury, arsenic, and lead).^75^ Geo-mapping of hazardous sites found that a disproportionate number of towns overburdened by toxic sources were also home to high proportions of BIPOC, a robust positive predictor of hazardous waste site locations.^76^

*ED Actionable Steps:* Increase syndromic surveillance collaborations with public health departments for early detection and community notification of hazardous conditions, and advocate for targeted policy interventions by highlighting the harmful health impacts on local communities.

#### Long-bone Fractures

Black and Hispanic patients are less likely to receive opioid analgesia for acute pain in the ED and opioid prescriptions at discharge compared to White counterparts.^77^^–^^79^ Research shows that although average pain scores do not differ between White and non-White patients with long-bone fractures (LBF), White patients are more likely to receive opiates (70% vs 50%, *P* < 0.001).^78^ Among children presenting for ED management of LBF, the data is similar: Black and Hispanic children were less likely to receive opioid analgesics (aOR 0.86, 95% CI 077–0.95 and 0.86, 95% CI 0.76–0.96, respectively) and less likely to achieve optimal pain reduction (aOR 0.78, 95% CI 0.67–0.90 and 0.80, 95% CI 0.67–0.95, respectively).^80^

*ED Actionable Steps:* Consider protocolized ED triage and early management of LBF, including adequate analgesia dosing schedules.

#### Firearm Injuries

Firearm violence is a public health epidemic in the US. In 2018, firearms were the leading method of homicide and suicide, major causes of premature death. Per the CDC, 39,707 Americans died from firearm violence in 2019, averaging 109 deaths per day and comprising 60% suicides, 35% homicides, and 1.4% law enforcement interventions.^81^ While most firearm suicide deaths impact Whites and American Indian/Alaska Natives, homicides disproportionately plague Black Americans. In 2018, firearm homicides were highest among Blacks. Black males and females aged 20–34 years died by firearm homicide at nearly 17 times higher and nearly six times higher rates than their White counterparts, respectively. Among youth aged 0–19, Black males had the highest firearm homicide rate at 14 times higher than their White peers. American Indian/Alaska Native male youth had the second highest youth homicide rate. Black males are disproportionately killed by law enforcement intervention with firearms at a rate 1.71 times that of non-Hispanic White males.^82^

*ED Actionable Steps:* Remain informed of local firearm injury statistics and advocate for adequate policy responses by highlighting the harmful health impacts on local communities.

## DISCUSSION

Across clinical pathology interests and in almost every area studied, BIPOC communities experience worse patient care and health outcomes. Contrary to historical medical teachings, there is no biological evidence for the concept of race as a genomic human subspecies to explain health disparities.^83^^,^^84^ Rather, it is the social interpretation of people in a race-conscious society that disparately impacts health.^85^ The system of structuring opportunity and assigning value, based on assumptions about groups of people with certain physical attributes, systematically privileges some while disadvantaging others and undergirds the deadly problem of structural racism. Compounding the well-recognized theory of resource deprivation among racially/ethnically segregated communities (eg, quality primary education, adequate housing, green space) is socioenvironmental theory, which points to acts of commission that inequitably pose health risks (eg, air pollution,^72^^,^^73^ toxic waste,^74^^–^^76^ and fast- food,^62^^–^^64^ alcohol,^86^ and tobacco outlets^87^).

Physicians must acknowledge the insidious health threat that implicit biases and structural racism pose. Disproportionate levels of socioeconomic disadvantage, social vulnerability, and poor health outcomes are manifestations of long-established and deeply entrenched racial segregation and racial deprivation. One could argue that the adverse health effects of structural racism over the centuries have created a far greater public health crisis than the COVID-19 pandemic, and yet beyond their identification, they have not received the attention they demand. Perhaps, in future years, our collective response to the volatile sociopolitical events of the last five years will be viewed as the force that changed the narrative. Many academic medical centers have created executive positions focused on equity, diversity, and inclusion and have worked to implement educational curricula aimed at dismantling structural racism.^88^

The question that remains today— how do we as individuals and collectively as an institution and specialty best advance social justice and health equity? —demands thoughtful actions and transformative policies. A recent scoping review found 37 published intervention papers with only a third including empirical research.^89^ Clearly, the implementation science behind this massive multi-pronged process will take time to develop,^90^ but there appears to be sufficient direction to propose potential actionable steps (Table 2) and practice recommendations.

**Table 2.** Potential actionable steps for emergency physicians.

**Communicable diseases**	1. DPH-funded, community partnerships for pop-up screening clinics in the community designed to provide rapid testing and counseling regarding treatment initiation for HIV, hepatitis C, and STIs. 2. DPH-funded, community partnerships for pop-up vaccination clinics in the community designed to provide testing, vaccination, and transmission-mitigation education in the community. 3. Self-guided education and peer education about the increased risk for severe COVID-19 and other respiratory and diarrheal morbidity and mortality among ethnic and racially diverse populations. 4. Empower patients with a thorough understanding of communicable diseases, including natural course of illness, methods of transmission, transmission prevention, and reasons for returning; discharge counseling techniques may include discharge nursing teach-back or read-back of instructions. 5. DPH-medical-community partnerships designed to focus efforts in areas of high transmission risk when planning resource distribution of testing, treatment, and vaccination supplies related to COVID-19 and other pandemic-related illnesses.
**Non-communicable conditions**	1. Educate EPs about long-standing racial and ethnic gaps in ED-based care and health outcomes; and promote opportunities for implicit bias training. 2. Develop equity metrics, monitor clinical performance data on quality measures, identify inequities in clinical and research, and implement process and policy changes to close disparity gaps. 3. Support health equity initiatives at the individual, departmental, and organizational levels that aim to educate patients about certain medical conditions (eg, hypertension, diabetes), early warning signs of serious complications (eg, acute coronary syndrome, renal failure), and available treatment options; educational strategies may involve smart documents and waiting room video educational modules. 4. Support and partner with existing patient care navigator and community health worker programs to engage patients beyond the index ED visit and ensure medication and treatment plan adherence, outpatient follow-up scheduling, and regular assessments of any barriers to disease control. 5. Partner with local community organizations designed to promote healthy lifestyle (eg, smoking cessation, nutritional food planning, local farm food collaborative, reduced-fee gym memberships, etc).
**Injuries**	1. Consider the potential environmental determinants of lung inflammation and injury in BIPOC patients with difficult-to-control asthma symptoms; educate patients about PM and its relationship to asthma and counsel them on preventative measures and importance of maintenance medication adherence. 2. Support and advocate for state and federal legislation and policy aimed at prevention of toxic waste dumping, containment efforts, periodic testing of soil and water supplies, increased testing for environmental exposures among communities living in high-risk exposure areas, and investment in industrial waste decontamination, safer housing, and quality medical care for affected communities. 3. Self-guided education and peer education about the signs and symptoms of toxicity due to common hazardous waste contaminants, and the available treatments. 4. Provide opioid analgesia for acute severe pain in the ED based on likely diagnosis, objective measures of pain, and optimal pain reduction (at least a 2-level reduction in pain score for initial treatment). 5. Support epidemiologic and narrative research of firearm violence, both nonfatal injuries and deaths, to better understand risk and protective factors as the basis for intervention. 6. Use the results of epidemiologic and narrative research to partner with communities to develop and implement effective interventions especially targeted at high-risk youth and young adults of color. 7. Partner with existing programs and personnel that have operated trauma center resources for community and firearm violence to extend their inpatient work to reach a greater proportion of those in need by developing and implementing ED protocols to identify, counsel, and refer at-risk populations. 8. Educate EPs on the effective counseling of populations at disproportionate risk for community and firearm violence and incorporate smart discharge phrases into the electronic health record system. 9. Develop strong collaborations with community groups and social services to whom the ED could transition primary and secondary prevention; incorporate these referrals into discharge materials. 10. Encourage state and federal legislation and policy aimed at decreasing firearm homicides and nonfatal injuries (eg, decrease access to illegal firearms, increase federal funding for research on firearm violence, decrease the production of violent video games and media and replace them with games in which the protagonist must save lives rather than kill to win).

*BIPOC*, Black, indigenous and people of color; *DPH*, Department of Public Health; *ED*, emergency department; *EP*, emergency physician; *PM*, particulate matter; *STIs*, sexually transmitted infections.

## LIMITATIONS

As critical reviews focus on advancing thought through conceptual innovation following an analysis of the literature, the methodology, by design, does not necessitate an exhaustive comprehensive review of the literature nor the same systematicity and quality assessment as in other more structured review approaches.^11^ Additionally, the objective of the conceptual product of a critical review is to propose a new phase of research within the field in question,^11^ and as a result, the actionable steps and practice recommendations made have yet to be proven effective but instead serve as a starting point for a new phase of implementation science.

## CONCLUSION

The suggested actionable steps and following practice recommendations constitute the conceptual product of this critical review, demanding a new phase of implementation and evaluation research that identifies effective strategies and best practices for mitigating racial health inequities. Emergency physicians, as individuals and organizational leaders, can act from several positions in the social structure:

A. Societal members
  1. Participate in local, state, and federal government forums advocating for health through resources and advantages historically inaccessible to BIPOC:
    a. Affordable, safe housing
    b. Food security (ie, sufficient, safe, and nutritious sustenance)
    c. Firearm safety, neighborhood safety, and support for survivors of violence
    d. Health-promoting lifestyle (eg, green space and density restrictions on fast-food, tobacco, and alcohol outlets)
    e. Comprehensive community health centers with expanded hours of operations
  2. Develop meaningful individual and organizational partnerships with antiracist stakeholders and communities (ie, Black Lives Matter, White Coats for Black Lives, etc).
  3. Engage leadership and representatives of first responder agencies in upholding the value of every human life.
B. Stewards of medicine
  1. Engage medical leadership in changing organizational culture to one that consistently prioritizes equity, addresses inequities in clinical and professional spaces, and recognizes the systematic advantage of privilege.
  2. Create permanent positions accountable to equity, diversity, and inclusion initiatives^91^ and ensure core leadership articulates diversity as an institutional priority and dialogues constructively with all relevant stakeholders.^92^
  3. Increase BIPOC representation within the pipeline and across all organizational strata.^93^
  4. Identify racial disparities and their sources within the system, conduct root cause analyses, and implement strategies to remedy inequities.^94^ Describe, document, and proactively work to mitigate the health impact of racism.^95^
  5. Draft policies and enforce protocols for dealing with race-based aggression by patients and other staff.
  6. Educate medical personnel through multimodal continuous medical education on trauma-informed care, anti-racism practice, and cultural humility.^96^
  7. Offer medical education curricula and periodic trainings for students, residents, community physicians, and faculty that include the following:^93^^,^^97^
    a. SDoH: Although the prospective, patient-oriented outcome is sparse, many medical schools and residency programs have adopted SDoH curriculum, which may lead to measurable changes in the future^98^ and is a stated priority of the Institute of Medicine.^99^ Comprehensive training materials are free and available on the web.^100^
    b. Cultural humility training to address implicit bias, stereotypes, and prejudice.^101^
    c. Anti-racism and trauma-informed care training to improve patient care communication and bedside skills.
  8. Evaluate the impact of educational programs on patient care and health outcomes to curate efforts.^102^ Disseminate evidence-based best practices.
  9. Endeavor as an institution and specialty to eliminate racialized conceptions of disease susceptibility (eg, casting Blacks as innately diseased and dehumanizing their suffering).^103^
C. ED staff
  1. Develop equity metrics, monitor clinical performance data, identify clinical and research gaps, and implement process and policy changes to eliminate health disparities.
  2. Abandon the practice of stating the patient’s race in the narrative of the history and physical as it has minimal benefit, risks introducing bias, and is offensive to minority physicians.^104^
  3. Cease the use of correction formulas that use race as a proxy for pathology when their use furthers health inequities.^105^
  4. Make deliberate efforts to treat racial groups similarly on individual and population levels as a concrete first step in ameliorating racial health disparities. Although physicians undoubtedly carry implicit racial biases equal to the general population, there is some evidence that emergency physicians show less implicit racial bias than the general population.^106^
  5. Address racist patient attitudes professionally even when these cause moral distress.^107^ Addressing racism and attempting to rebuild therapeutic alliances is part of the leadership and professionalism that emergency physicians must emulate.
D. Hospital executives
- Institutional leaders must assure appropriate ED ancillary staffing and address hospital policies (eg, inpatient census levels, direct and transfer admissions) that result in ED crowding, medical error, morbidity and mortality, and staff demoralization.^108^ Emergency physicians are experts in rapid cognition or thin-slicing, but with that practice comes the expression of latent stereotypes and biases that require a deliberate “bias-check” pause to better understand the patient and, thus, achieve better outcomes.^109^ Research has demonstrated that overstressing physicians beyond reasonable levels is associated with increases in implicit bias.^110^
E. Clinical caregivers
  1. Employ a trauma-informed care approach with individual patient interactions.^111^ The BIPOC communities suffer under the pervasiveness of historical and personal trauma as well as the psychological trauma inflicted by law enforcement killings of unarmed Blacks.^112^ Moreover, BIPOC minorities are exposed daily to stressful and traumatic events at much great rates than the general population.^113^ To adopt the trauma-informed care framework:
    a. Abandon power imbalances common in traditional, paternalistic doctor-patient dynamics.
    b. Empower patients to be partners in treatment decisions.
    c. Offer patients validation, explanation, and choice.
    d. Practice cultural humility, an orientation to care that is based on self-reflexivity, appreciation of patients' lay expertise, openness to sharing power and knowledge with patients, and desire to learn from patients.^114^
  2. Recognize and counter potentially racist clinical decisions by doing the following:
    a. Follow evidence-based race-blind admission and surgical criteria.
    b. Provide professional peer-to-peer feedback with coaching on delivery of difficult conversations.^115^
    c. Build race-blind analgesia protocols.^116^
    d. Create policies to address interprofessional microaggressions and patient-to-clinician racism. Micro- and macroaggressions contribute to burnout and must be combated to ensure inclusion and career longevity.^117^^,^^118^

In conclusion, from a medical standpoint, there is only one race—the human race—and we must recognize and counter our implicit biases. As fellow humans, we must acknowledge that structural racism drives health inequities, and as emergency physicians we can choose to address it by employing any or all the actions and recommendations proposed herein.
